# Selective dopaminergic vulnerability in Parkinson’s disease: new insights into the role of DAT

**DOI:** 10.3389/fnins.2023.1219441

**Published:** 2023-08-24

**Authors:** Maged M. Harraz

**Affiliations:** ^1^Department of Psychiatry, University of Maryland School of Medicine, Baltimore, MD, United States; ^2^Department of Pharmacology, University of Maryland School of Medicine, Baltimore, MD, United States

**Keywords:** dopamine transporter, Parkinson’s disease, autophagy lysosome pathway, dopamine toxicity, nigral degeneration

## Abstract

One of the hallmarks of Parkinson’s disease (PD) is the progressive loss of dopaminergic neurons and associated dopamine depletion. Several mechanisms, previously considered in isolation, have been proposed to contribute to the pathophysiology of dopaminergic degeneration: dopamine oxidation-mediated neurotoxicity, high dopamine transporter (DAT) expression density per neuron, and autophagy-lysosome pathway (ALP) dysfunction. However, the interrelationships among these mechanisms remained unclear. Our recent research bridges this gap, recognizing autophagy as a novel dopamine homeostasis regulator, unifying these concepts. I propose that autophagy modulates dopamine reuptake by selectively degrading DAT. In PD, ALP dysfunction could increase DAT density per neuron, and enhance dopamine reuptake, oxidation, and neurotoxicity, potentially contributing to the progressive loss of dopaminergic neurons. This integrated understanding may provide a more comprehensive view of aspects of PD pathophysiology and opens new avenues for therapeutic interventions.

## Introduction

Parkinson’s disease (PD) is a progressive neurodegenerative movement disorder. While mainly characterized by motor syndrome, PD is associated with non-motor symptoms (NMS) in almost all patients. More than 10 million primarily older adults worldwide suffer from PD. The most common symptom associated with PD is tremor, which typically begins on one side before eventually affecting both sides over time. Other symptoms may include muscle rigidity (stiffness), gait disturbances (difficulty walking), slowness/decreased mobility, impaired speech/swallowing problems, depression/anxiety disorders, hyposmia, constipation, urinary dysfunction, orthostatic hypotension, pain, sleep disturbances, and cognitive decline, including dementia or psychosis later in life if left untreated for too long. While there is no cure for PD, symptomatic dopaminergic therapy helps improve the quality of life for those with the disease. PD is characterized by the presence of certain hallmarks in its pathology. These include progressive selective loss of dopaminergic neurons in the midbrain substantia nigra pars compacta (SNpc); Lewy bodies containing aggregates of α-synuclein protein; gliosis or inflammation around affected areas; and a dramatic decrease in dopamine, a neurotransmitter associated with movement and reward. While the motor syndrome of PD is related to nigral degeneration and dopamine depletion, NMS may be linked to the degeneration of other neural types, including the peripheral autonomic nervous system (Girault and Greengard, 2004; Tolosa et al., 2021; Haider et al., 2023). PD is a multifactorial disease with genetic and environmental factors that converge on common pathophysiological processes (Simon et al., 2020). Dopaminergic therapy can improve motor symptoms but does not alter the underlying progressive neurodegeneration. As such, there is a need to develop disease-modifying therapeutic strategies that target underlying pathophysiological mechanisms to slow or arrest the progression of PD (Murakami et al., 2023).

**Figure 1:**
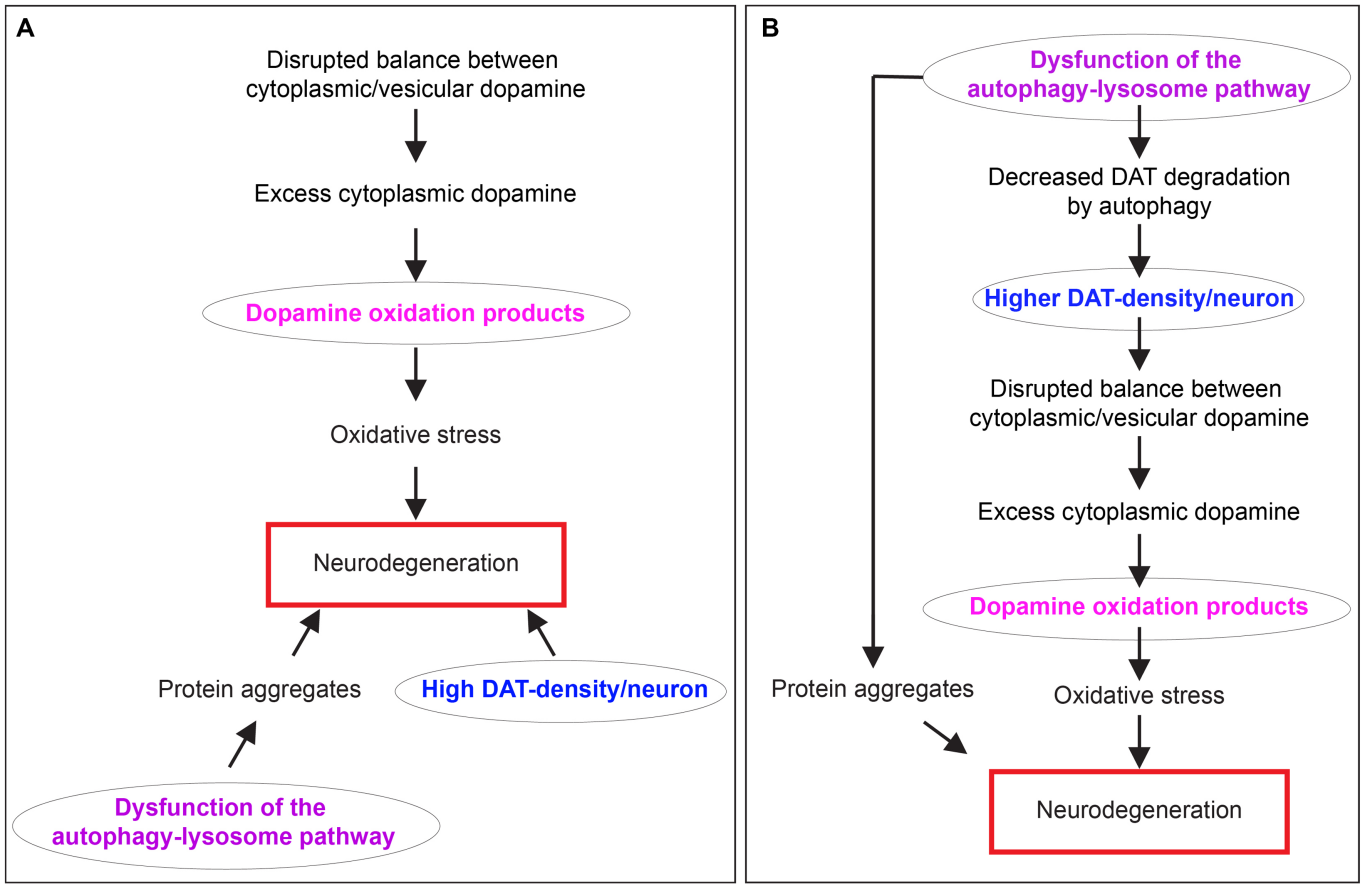
The relationship between dopamine oxidation, DAT expression density, and autophagy-lysosome pathway dysfunction in neurodegeneration in PD. **(A)** A diagram showing three separate mechanisms are proposed to contribute to neurodegeneration in PD. **(B)** A diagram depicting the interrelationship between dopamine oxidation, DAT expression density, and autophagy-lysosome pathway dysfunction in PD neurodegeneration unified by DAT autophagy.

The molecular pathogenesis of PD involves a complex interplay of several factors, such as impaired mitochondria, protein homeostasis, autophagy-lysosome pathway (ALP) dysfunction at the synapse, trafficking of the dopamine transporter (DAT), DA toxicity, oxidative stress, disruptions in synaptic vesicle endocytosis, autonomous pacemaking, calcium homeostasis imbalance, iron-content, extensive axonal arborization size, prion-like α-synuclein transmission, and neuroinflammation. The reader is referred to several review articles that discuss these pathophysiological mechanisms in detail (Lohr et al., 2017; Giguère et al., 2018; Nguyen et al., 2019; Lu et al., 2020; Bu et al., 2021). Acknowledging that neuronal loss in PD cannot be explained by one pathway, I focus on uniting three of the above-mentioned pathophysiological mechanisms: (1) Dysfunction of the ALP. (2) High expression density of DAT per neuron, which strongly correlates with the extent of dopaminergic neuron loss. (3) Dopamine oxidation mediated neurotoxicity. Several groups characterized each of these mechanisms for their potential role in the pathophysiology of PD. Still, there was no apparent connection to link them together (Figure 1A). This minireview unites these concepts and clarifies their interrelationships considering our recent discovery of autophagy as a novel mechanism for controlling dopamine homeostasis (Figure 1B). Here, I discuss how dysregulated autophagic degradation of DAT could be considered a contributing element in mediating selective dopaminergic degeneration in PD through DA toxicity.

## The autophagy lysosome pathway in PD

The ALP plays a crucial role in the pathogenesis of PD. Autophagy, or self-eating, is a process by which cells degrade and recycle their cellular components, such as organelles, proteins, lipids, and nucleic acids, for use in metabolic processes. The autophagic process is regulated by several molecular pathways involving the lysosomes and other organelles, including the endoplasmic reticulum and mitochondria (Fleming et al., 2022). The lysosomes are the destination for autophagic cargo for degradation. There are three types of autophagy:

1. Macroautophagy: characterized by the formation of a double membrane organelle, the autophagosome, which forms around its cargo and transports it to the lysosome. There are two types of macroautophagy in terms of cargo recognition: selective and non-selective. Relevant examples of selective macroautophagy include mitophagy (mitochondria) and aggrephagy (protein aggregates), among many others (Galluzzi et al., 2017).
2. Microautophagy: characterized by the lysosome’s direct internalization of small cytoplasmic cargo. Microautophagy can also be selective and non-selective (Wang et al., 2023).
3. Chaperone-mediated autophagy (CMA): characterized by selective recognition of cytoplasmic proteins harboring a KFERQ or KFERQ-like motif by chaperones that escort them to the lysosome where they undergo translocation through the lysosomal membrane to its lumen for degradation (Kaushik and Cuervo, 2012).

In PD, the ALP pathway has been found to be dysregulated in both familial (Beilina et al., 2014) and sporadic (Kabuta and Wada, 2008; Wu et al., 2008, 2011; Lonskaya et al., 2013; Beilina et al., 2014; Moors et al., 2019) forms of the disease, leading to the accumulation of toxic protein aggregates within neurons resulting in neuronal death or dysfunction.

Several genes related to ALP have been implicated in PD due to their mutations. These genes have roles in various aspects of the pathway, from regulating autophagy initiation to the proper functioning of lysosomes. Here are a few examples of some key genes:

- Leucine-rich repeat kinase 2 (*LRRK2*) mutations are one of the most common genetic causes of autosomal dominant PD. The LRRK2 protein is involved in various cellular processes, including autophagy and endolysosomal functions. Mutations can lead to impaired autophagy and increased susceptibility to PD (Gómez-Suaga and Hilfiker, 2012; Orenstein et al., 2013; Ysselstein et al., 2019).
- *GBA* mutations are the most frequent genetic risk factor for PD. *GBA* encodes the lysosomal enzyme glucocerebrosidase, which is involved in the breakdown of glucosylceramide. Reduced glucocerebrosidase activity is associated with impaired lysosomal function and α-synuclein accumulation (Pang et al., 2022; Pradas and Martinez-Vicente, 2023).
- Vacuolar protein sorting ortholog 35 (VPS35) is a retromer complex component. VPS35 mutations have been linked to autosomal dominant PD. VPS35/retromer complex is essential for the retrieving specific membrane proteins from endosomes to the trans-Golgi network, thereby maintaining lysosomal function (Zavodszky et al., 2014; Cui et al., 2021).
- ATPase 13A2 (ATP13A2), also known as PARK9, is a lysosomal P-type ATPase. Mutations in this gene are associated with a rare form of early-onset parkinsonism (Kufor-Rakeb syndrome). ATP13A2 is involved in maintaining lysosomal pH and manganese homeostasis, and its dysfunction contributes to impaired autophagy (Dhanushkodi et al., 2023).
- PTEN-induced putative kinase 1 (PINK1) mutations are associated with autosomal recessive early-onset PD. PINK1 is a mitochondrial kinase that plays a key role in mitophagy, a selective form of autophagy responsible for removing damaged mitochondria (Jones, 2010).
- Parkin RBR E3 Ubiquitin Protein Ligase (PARKIN) mutations are also linked to autosomal recessive early-onset PD. PARKIN functions as an E3 ubiquitin ligase and works with PINK1 in the mitophagy pathway, promoting the clearance of damaged mitochondria (Herman and Moussa, 2011).
- Protein deglycase DJ-1, also known as Parkinson’s disease protein 7 (DJ-1), mutations are implicated in autosomal recessive early-onset PD. DJ-1 has multiple roles in the cell, including acting as a redox-sensitive chaperone and a sensor for oxidative stress. DJ-1 is also involved in regulating autophagy and mitophagy (Wang et al., 2016).
- Auxilin (DNAJC6/PARK19) is a cofactor for uncoating clathrin-coated vesicles for the heat shock cognate protein-70 (Hsc70) (Nguyen et al., 2019). Its loss-of-function mutations cause early-onset Parkinson’s disease (PD) (Olgiati et al., 2016; Ng et al., 2020), and its mutations also occur in late-onset PD (Gialluisi et al., 2021).

Most PD cases are sporadic and not linked to known genetic mutations. Sporadic PD is thought to result from a combination of genetic and environmental factors, with aging being the most significant risk factor. Yet, recent studies implicate a significant role of heredity in PD that was not previously appreciated, suggesting that 27 to 60% is due to genetic factors (Hamza and Payami, 2010; Do et al., 2011; Keller et al., 2012). Many genes and genetic loci are now linked to the risk for PD using modern genetic methods. Many studies suggest that ALP might be involved in sporadic PD (Orenstein et al., 2013; Zavodszky et al., 2014; Ysselstein et al., 2019; Cui et al., 2021; Pang et al., 2022; Shrivastava et al., 2022; Pradas and Martinez-Vicente, 2023). A recent meta-analysis of genome-wide association studies (GWAS), interrogating data from >13,000 PD patients and > 95,000 controls, identified 26 genetic loci across the genome associated with PD. Intriguingly, 16 out of these 26 genetic loci have 18 genes nearby that modulate ALP, namely *SYT11* (Bento et al., 2016), *RAB7L1* (Kuwahara et al., 2016; Pradhan et al., 2018; Shrivastava et al., 2022), *NUCKS1* (Zhao et al., 2020), *GBA* (Murphy and Halliday, 2014; Pang et al., 2022; Pradas and Martinez-Vicente, 2023), *SIPA1L2* (Andres-Alonso et al., 2019), *TMEM163* (Cuajungco et al., 2014; Cuajungco and Kiselyov, 2017), *STK39* (Gallolu Kankanamalage et al., 2016, 2017; He et al., 2022), *LAMP3* (Tanaka et al., 2022), *TMEM175* (Jinn et al., 2017; Hu et al., 2022), *GAK* (Miyazaki et al., 2021; Munson et al., 2021), *SCARB2* (Velayati et al., 2011), *GPNMB* (Robinet et al., 2021; Zhu et al., 2022), *VPS13C* (Cai et al., 2022; Hancock-Cerutti et al., 2022), *RIT2* (Obergasteiger et al., 2023), *DDRGK1* (Cao et al., 2021), *SNCA* (Sampaio-Marques et al., 2012; Song et al., 2014), *LRRK2* (Gómez-Suaga and Hilfiker, 2012; Orenstein et al., 2013; Ysselstein et al., 2019), and *MAPT* (Zhu et al., 2017; Feng et al., 2020).

## Dopaminergic degeneration in PD, potential role of the dopamine transporter

DAT is a membrane-spanning protein that plays a principal role in regulating dopaminergic neurotransmission mainly through modulating the availability and duration of action of dopamine. It is responsible for the reuptake of released dopamine back into presynaptic neurons (Bu et al., 2021). Multiple lines of evidence support the theory that DAT is involved in selective dopaminergic neurotoxicity and degeneration in PD (Storch et al., 2004). Studies have demonstrated a correlation between DAT expression levels and the extent of neurodegeneration, as well as an association between genetic polymorphisms of DAT and PD risk. Additionally, animal models have shown increased vulnerability to toxins with higher concentrations of DAT in striatal regions affected by PD pathology. These findings suggest that dysregulation or malfunctioning of DAT may contribute to neuronal death associated with PD pathogenesis.

## DAT density per neuron correlates with neurodegeneration vulnerability in PD

Consistent with a role for DAT in the pathophysiology of PD, the positive correlation between specific dopaminergic neuron subtypes’ vulnerability with the relative DAT expression levels. Hence, hypothalamic dopaminergic neurons are spared in PD, while the ventral tegmental area (VTA) dopaminergic neurons are moderately affected. The dopaminergic neurons in SNpc are most affected in PD. In contrast, the dopaminergic neurons spared in PD patients’ postmortem tissues show lower DAT expression per neuron than controls. For example, Uhl et al. reported that DAT mRNA per neuron is higher in SNpc compared to the adjacent nucleus paranigralis of the ventral tegmental area (VTA) in neurologically normal study subjects. Surviving SNpc neurons in age-matched PD patients is almost half that in controls (Uhl et al., 1994). The same finding was reproduced by other groups (Uhl et al., 1994; Joyce et al., 1997). These findings suggest the selective vulnerability of neurons expressing high levels of DAT in PD. An alternative explanation could be a compensatory downregulation of DAT mRNA levels in the low-expressing neurons in PD. However, this alternative scenario is less likely since the same differential expression of DAT mRNA was documented in controls across species (Sanghera et al., 1994; Haber et al., 1995; Bannon and Whitty, 1997; Afonso-Oramas et al., 2009). Along the same lines, a recent meta-analysis study suggests that the *SLC6A3* 10R variant, associated with relatively lower expression activity of DAT, may be a protective factor in susceptibility to PD (Zeng et al., 2021).

Consistent with the observations mentioned above, preclinical evidence demonstrates that ectopic or elevated DAT expression is sufficient to induce neurodegeneration. Chen et al. generated a Tet-inducible forebrain DAT-transgenic mouse model under the calcium/calmodulin-dependent protein kinase type II alpha (CaMKIIα) promoter. CamDAT mice spontaneously develop motor dysfunctions and progressive neurodegeneration in the striatum, which l-DOPA accelerates (Chen et al., 2008). Masoud et al. showed that overexpression of DAT in dopaminergic neurons (DAT-transgenic mice) shows impromptu loss of midbrain dopaminergic neurons and increased oxidative stress. DAT protein levels are elevated in DAT-tg mice only in dopaminergic neurons. DAT-tg mice show a < 50% increase in dopamine uptake rate, increased metabolite/dopamine ratios, and diminished striatal VMAT2 protein expression. Interestingly, DAT-tg mice show l-DOPA reversible motor deficits (Salahpour et al., 2008; Masoud et al., 2015). These data show that a 30% DAT activity increase in dopaminergic neurons is sufficient to induce oxidative stress and spontaneous dopaminergic neurodegeneration *in vivo*.

## Evidence showing increased DAT levels in DA neurons in PD or PD models

One of the hallmarks of PD progression is the reduction of DAT levels in the brain, which is the direct result of the loss of DA terminals. However, several studies observed increased DAT levels without or preceding dopaminergic neurodegeneration. Here I list examples of these studies.

## Clinical evidence

A cross-sectional analysis in the subset of non-manifesting carriers of *LRRK2* and *GBA* mutations enrolled into the Parkinson’s Progression Markers Initiative (PPMI) focused on 123-I Ioflupane DAT imaging reported that non-manifesting carriers of *GBA* mutation had increased DAT binding in all striatal regions compared with healthy controls and non-manifesting carriers of the *LRRK2* mutation (Simuni et al., 2020). Interestingly, many lines of evidence show that glucocerebrosidase (the enzyme encoded by the *GBA* gene) regulates autophagy (Bento et al., 2016; Lu et al., 2020; Kuo et al., 2022; Navarro-Romero et al., 2022). The biological mechanism driving increased DAT binding in non-manifesting carriers of *GBA* mutations remains unclear. Since PPMI is a longitudinal study, it will be important to determine whether the observed up-regulation of DAT in *GBA* non-manifesting carriers is associated with an increased risk of PD.

In a smaller clinical study, a significant increase in striatal DAT was observed in non-manifesting carriers of the 22q11.2 mutation, which is associated with an age-related increased risk of PD, suggesting that a hyperdopaminergic mechanism may be linked to manifestations of PD and its pathogenesis (Butcher et al., 2017).

## Preclinical evidence

Data from non-human primates show a robust increase in DAT expression in monkeys treated with α-synuclein delivered via Adeno-associated virus (AAV) as assessed by 123I-PE2I single-photon emission computerized tomography scans and confirmed by post-mortem immunohistochemical analyses. This non-human primate model induces Parkinson’s disease-like pathology, including α-synuclein aggregation with pathological properties such as amyloid-like Lewy body pathology in PD brains and nigral neuronal degeneration (Chu et al., 2019). Similarly, AAV-mediated delivery of human A53T α-synuclein to the rat SN leads to behavioral deficits and PD-like nigro-striatal degeneration in a dose-and time-dependent manner. Human A53T α-synuclein expression in the SN significantly increased striatal DAT and DA turnover 3 weeks post-AAV injection before overt pathology. By 6 weeks post-AAV injection, SN DA neurons, striatal DA, TH, and DAT were reduced, and a sustained behavioral deficit was observed (Koprich et al., 2011). Furthermore, α-synuclein overexpressing (SNCA-OVX) mice show increased DA uptake and upregulation of membrane DAT levels as assessed by *in situ* autoradiography and DAT-immunostaining, correlating with α-synuclein levels (Threlfell et al., 2021). Further, Bellucci et al. reported a significant increase in DAT levels in the brain of the truncated α-synuclein (SYN120) mice (Bellucci et al., 2011).

LRRK2 G2019S mutant knock-in (GKI) mice show a significant increase in striatal DAT protein as assessed by western blot (Longo et al., 2017; Volta et al., 2017; Domenicale et al., 2022) and dopamine uptake (Longo et al., 2017; Domenicale et al., 2022). These changes to DAT levels are observed in LRRK2 G2019S mutants but not LRRK2 kinase-dead or knock-out mice (Domenicale et al., 2022).

In DJ-1 KO mice, multiple groups found no difference in DAT mRNA or total DAT protein levels in striatal lysates (Chen et al., 2005; Goldberg et al., 2005; Manning-Boğ et al., 2007). However, the same studies indicate an increase in surface DAT, which is the functional pool of the protein. Young adult DJ-1 KO mice (4 months) show faster DA uptake (V_max_) in the dorsal but not ventral caudate-putamen (Chen et al., 2005), indicating a significant increase in surface DAT since the velocity of DA uptake reflects the functional number of DAT molecules on the cell surface (Daniels and Amara, 1999; Melikian and Buckley, 1999; Sorkina et al., 2005). Similarly, Goldberg et al. reported increased DA reuptake in the dorsal striatum of 3 months old DJ-1 KO mice (Goldberg et al., 2005). Manning-Boğ et al. also reported increased DAT protein levels in the synaptosomal fraction of DJ-1 young adult mice striata (3–4 months). This increase in cell surface DAT was confirmed by an increased active DAT as assessed by [125I]-RTI-121 binding and increased [3H]-DA uptake in striatal synaptosomes (Manning-Boğ et al., 2007).

Auxilin KO mice recapitulate all the critical hallmarks of PD; age-dependent α-synuclein pathology, selective dopaminergic degeneration, astrogliosis, and microgliosis leading to motor deficits that are reversed by L-DOPA treatment. Vidyadhara et al. demonstrate that the lack of auxilin leads to dopaminergic degeneration likely through three processes in the nigral nerve terminals: (Girault and Greengard, 2004) a dramatic upregulation of surface DAT in young mice before dopaminergic degeneration (3-month-old mice) (Haider et al., 2023), toxic accumulation of cytoplasmic DA before neurodegeneration, and (Tolosa et al., 2021) accumulation of autophagosomes in DA synaptic buttons at 3-months, which becomes worse later with the observation of dopaminergic degeneration (Vidyadhara et al., 2023). Interestingly, the auxilin KO mice replicate all three mechanisms united by DAT autophagy, as further explained below.

In summary, several studies show increased DAT levels before dopaminergic degeneration, suggesting that upregulation of DAT might be a part of an early pathological process in PD.

## Dopamine toxicity

Dopamine is an essential neurotransmitter that plays a critical role in various physiological processes, including reward, motivation, cognition, and motor function. Dopamine toxicity refers to the excessive accumulation of dopamine within the cytoplasm of dopaminergic neurons in the brain, which can lead to their dysfunction and death. Dysregulation of dopamine signaling is implicated in many neurological and psychiatric disorders, such as PD, schizophrenia, and addiction. The toxic effects of dopamine may involve oxidative stress, mitochondrial dysfunction, and the formation of toxic metabolites. In addition, the accumulation of abnormal protein aggregates in the neurons may contribute to their degeneration (Masato et al., 2019).

Dopamine synthesis, packaging in synaptic vesicles, and reuptake are tightly regulated to prevent its accumulation in the cytoplasm, which could be deleterious. The vesicular monoamine transporter 2 (VMAT2) quickly loads dopamine into synaptic vesicles after its synthesis in the cytoplasm or reuptake in the presynaptic terminals by DAT. In addition to dopamine sequestration, the acidic pH ~5.6 of synaptic vesicles stabilizes dopamine (Mani and Ryan, 2009). Dopamine oxidation results from enzymatic or autooxidation (Umek et al., 2018) and produces reactive oxygen species (ROS), such as hydrogen peroxide, hydroxyl radicals, and superoxide. Dopamine autooxidation produces reactive quinones, which can damage cellular macromolecules and impair neural processes (Stokes et al., 1999). Quinone generation is either spontaneous and could be accelerated by metal ions, such as iron or manganese, or is the product of specific enzyme-catalyzed reactions. Quinone-induced damage to macromolecules and oxidative stress could initiate cell death pathways contributing to neurodegeneration (Mor et al., 2019).

Several dopamine–quinone molecules can undergo oxidative ligation to α-synuclein, selectively inhibiting the protofibril-to-fibril conversion (Conway et al., 2001). α-synuclein protofibrils are likely the toxic form that disrupts neural homeostasis and induces neural cell death through various intracellular targets (Stefanis, 2012). α-synuclein protofibrils can permeabilize synaptic vesicles leading to leakage of stored dopamine into the cytoplasm, further worsening dopamine toxicity (Volles et al., 2001; Lashuel et al., 2002a,b; Volles and Lansbury, 2002).

Monoamine oxidase (MAO) enzymatically metabolizes cytoplasmic dopamine to 3,4-dihydroxyphenylacetaldehyde (DOPAL), a toxic metabolite (Burke et al., 2003). Under physiological conditions, this reaction also produces ROS, such as hydrogen peroxide and hydroxyl radicals. ROS production contributes to oxidative stress and lipid peroxidation inhibiting aldehyde dehydrogenase (ALDH), the main enzyme that eliminates DOPAL, which in turn leads to the accumulation of DOPAL, perpetuating a vicious cycle that contributes to dopaminergic neurodegeneration (Masato et al., 2019). DOPAL levels are elevated in PD postmortem putamen tissue. Further, VMAT2 activity is decreased by 89% and ALDH activity by 70%, likely leading to less synaptic vesicle uptake of cytosolic dopamine and decreased DOPAL detoxification (Goldstein et al., 2013; Pifl et al., 2014). The decrease in VMAT2 activity likely reflects DA neuron terminal loss in PD. On the other hand, VMAT2 deficiency in mice is sufficient to induce cytoplasmic DA toxicity, dopaminergic degeneration in SNpc, and l-DOPA-responsive motor deficits (Caudle et al., 2007). These lines of research inspired the “*Catecholaldehyde hypothesis*,” depicting the role of DOPAL in SNpc dopaminergic degeneration in PD (Masato et al., 2019).

## Autophagy selectively targets DAT for degradation

Recently, our studies have suggested that DAT can be selectively targeted for autophagic degradation (Harraz et al., 2021). Our findings show that cocaine, a potent psychostimulant and addictive substance, induces autophagy in neurons with extraordinary potency. Hence, cocaine induces neural autophagy at sub-nanomolar levels both *in vitro* and *in vivo*. The rapid induction of autophagy by cocaine is evident in tyrosine hydroxylase-positive nerve terminals located in the nucleus accumbens (NAc), a brain region implicated in reward, motivation, and addiction. Remarkably, this induction of autophagy can be detected as early as 3 min after systemic cocaine injection. Cocaine administration leads to DAT depletion in dopaminergic nerve terminals in the NAc. The depletion of DAT by cocaine in the nucleus accumbens nerve terminals has been detected using various experimental techniques. Western blot analysis of isolated nerve terminals demonstrates a reduction in DAT protein levels, while immunostaining of brain sections reveals decreased DAT expression in dopaminergic neurons. Furthermore, dopamine uptake activity in isolated nerve terminals, assessed using radiolabeled dopamine (3H-dopamine), shows a reduction in the maximum velocity of dopamine uptake, confirming the depletion of DAT from the nerve terminals’ membranes. Interestingly, pharmacological inhibition of autophagy reverses the depletion of DAT induced by cocaine. This suggests that cocaine-mediated autophagy plays a significant role in modulating DAT levels and dopaminergic neurotransmission (Harraz et al., 2021).

Selective degradation of DAT by autophagy represents a novel mechanism for regulating dopamine signaling and dopaminergic neurotransmission. By modulating DAT levels, autophagy can control dopamine reuptake, its availability in the synaptic cleft, and its potential accumulation in the dopaminergic neurons’ cytoplasm in case of sustained elevated dopamine reuptake. This has important implications for the strength and duration of dopaminergic signaling and dopamine neurotoxicity.

## The interplay of dopamine oxidation, DAT density per neuron, and autophagy-lysosome pathway dysfunction

The above-mentioned abnormal mechanisms have been proposed to contribute to PD pathophysiology. These include:

1. Dopamine oxidation-mediated neurotoxicity. The catechol structure of dopamine makes it particularly susceptible to enzymatic and non-enzymatic oxidation, generating dopamine quinones and other ROS. These oxidative species can damage cellular components, disrupt mitochondrial function, and initiate cell death pathways, contributing to the neurodegeneration seen in PD.
2. High expression density of DAT per neuron in SNpc. The extent of dopaminergic neuron loss in PD has been found to correlate strongly with the expression density of DAT per neuron. High DAT density could increase dopamine reuptake and intracellular accumulation, promoting dopamine oxidation and associated neurotoxicity. However, the precise role and regulation of DAT in PD pathogenesis have remained unclear.
3. Dysfunction of the autophagy-lysosome pathway. The ALP has also been implicated in PD. Dysregulation of this pathway can lead to the accumulation of damaged proteins and organelles, including potentially neurotoxic dopamine metabolites. Although the connection between autophagy dysfunction and PD has been established, its implications for dopamine homeostasis and DAT regulation were not fully understood.

However, until recently, these mechanisms have been studied in isolation, with no apparent connection to link them together.

## Unifying the concepts: autophagy as a regulator of dopamine homeostasis

**Figure 2:**
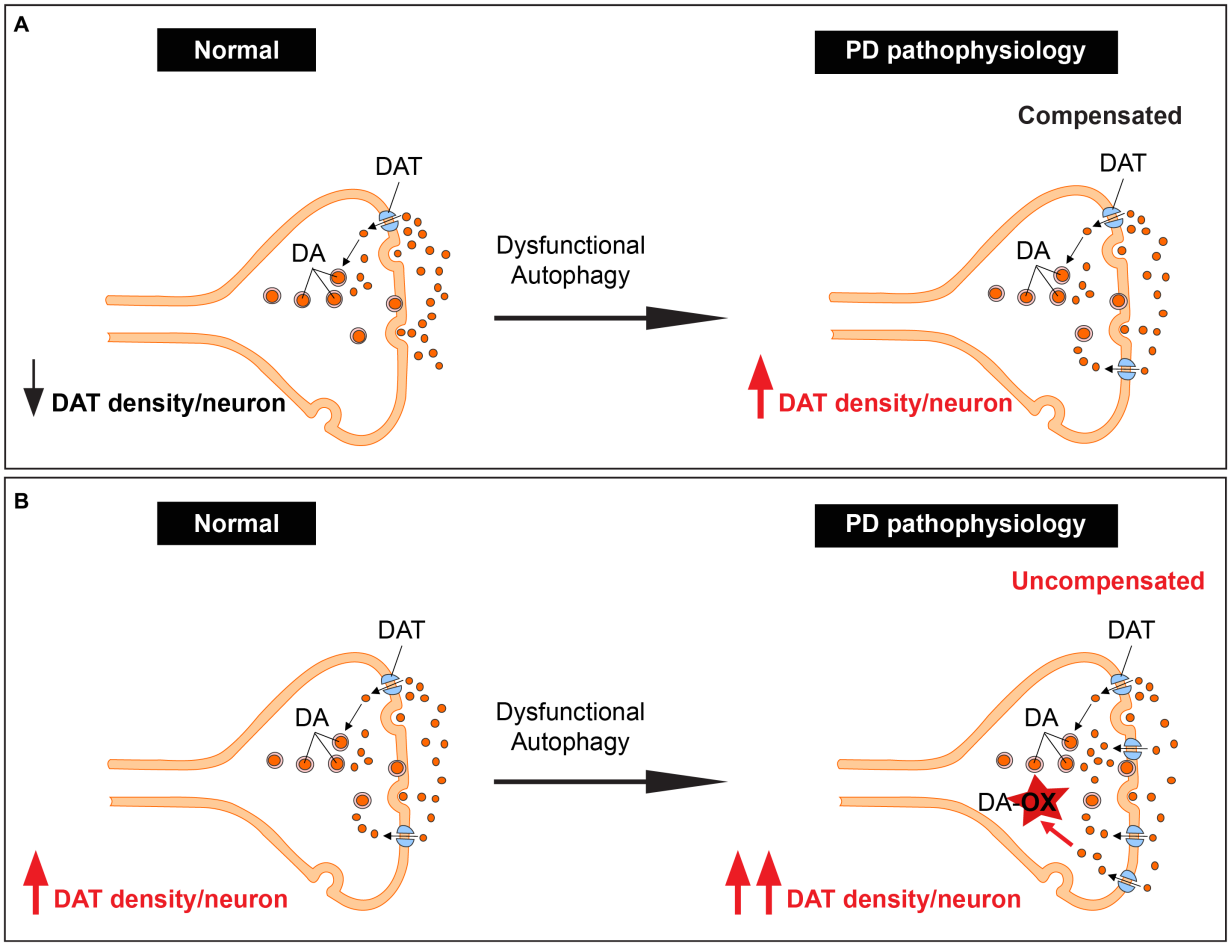
Unifying dopamine oxidation, DAT expression density, and autophagy-lysosome pathway dysfunction by autophagy regulation of dopamine homeostasis. **(A)** An illustration showing dopaminergic terminals with low basal DAT expression. These terminals are spared in PD since the increase in DAT levels due to dysfunction of the ALP leads to elevated but compensated dopamine reuptake. **(B)** An illustration showing dopaminergic terminals with high basal DAT expression. These terminals likely degenerate in PD since the increase in DAT levels due to dysfunction of the ALP leads to elevated uncompensated dopamine reuptake.

Our recent research has provided a unifying framework that integrates these previously separate mechanisms. We have discovered that autophagy is a novel mechanism for controlling dopamine homeostasis, directly linking autophagy-lysosome pathway dysfunction, DAT density, and dopamine oxidation. Autophagy declines with aging (Miki et al., 2018), an observation further complicated by ALP dysfunction in familial and sporadic PD (Kabuta and Wada, 2008; Beilina et al., 2014). Autophagic degradation of DAT is likely reduced due to PD pathology-mediated decline in autophagy. Chronic elevation of DAT density per neuron could lead to a sustained elevation in dopamine reuptake, disrupting the tight regulation of cytoplasmic vs. vesicular dopamine, leading to enhanced dopamine oxidation and neurotoxicity, especially in neurons with already preexisting high DAT expression. This interplay could contribute significantly to dopaminergic neuron loss and the progression of PD (Figures 2A,B).

## Conclusion

In conclusion, our research bridges the gap between three previously separate concepts in PD pathophysiology, providing a more comprehensive understanding of aspects of the disease’s pathogenesis. By recognizing autophagy as a novel regulator of dopamine homeostasis, a missing link is recognized between dysfunctional ALP, high expression density of DAT per neuron, dopamine oxidation-mediated neurotoxicity, and correlation with vulnerability of dopaminergic neurons in PD.

## Author contributions

The author confirms being the sole contributor of this work and has approved it for publication.

## Conflict of interest

The author declares that the research was conducted in the absence of any commercial or financial relationships that could be construed as a potential conflict of interest.

## Publisher’s note

All claims expressed in this article are solely those of the authors and do not necessarily represent those of their affiliated organizations, or those of the publisher, the editors and the reviewers. Any product that may be evaluated in this article, or claim that may be made by its manufacturer, is not guaranteed or endorsed by the publisher.
